# Prevalence of antibacterial resistance among hospitalized patients in Tigray, Ethiopia, 2021: A cross-sectional design

**DOI:** 10.1016/j.ijregi.2024.100477

**Published:** 2024-10-24

**Authors:** Migbnesh Gebremedhin Weledegebriel, Afewerki Tesfahunegn Nigusse, Hansa Haftu, Haylay Gebremeskel, Bisrat Tesfay Abera, Merhawit Atsbeha, Araya Gebreyesus Wasihun, Edris Ebrahim Edris, Kibra Hailu, Ephrem Solomon, Abrha Bsrat Hailu

**Affiliations:** 1 Department of Internal Medicine, College of Health Sciences, Mekelle University, Tigray, Northern Ethiopia; 2 Department of Epidemiology, College of Health of Science, Mekelle University, Tigray, Northern Ethiopia; 3 Department of Pediatrics and Child Health, College of Health Science, Mekelle University, Tigray, Northern Ethiopia; 4 Department of Medical Microbiology and Immunology, College of Health Science, Mekelle University, Tigray, Northern Ethiopia; 5 Ayder Comprehensive Specialized Hospital, Mekelle University, Tigray, Northern Ethiopia; 6 Legehar General Hospital, Addis Ababa, Ethiopia; 7 College of Veterinary Sciences, Mekelle University, Tigray, Northern Ethiopia

**Keywords:** Antibacterial resistant, Sepsis, AMR prevalence, Tigray

## Abstract

**Objectives:**

To describe the prevalence of antibacterial resistance among hospitalized patients in Tigray.

**Methods:**

A cross-sectional study design was applied in hospitalized septic medical patients in Ayder Comprehensive Specialized Hospital from March 2020 to January 2021. Socio-demographic, clinical, and laboratory-related data was collected, and descriptive analysis was done to present the result.

**Results:**

In this study, a total of 153 hospitalized septic patients were included with a median age of 30 years (interquartile range 22-49). A total of 187 different samples were collected from 153 participants of the blood and foci of infection, and bacterial isolates were found in 103 (55%) samples. A total of 70 (45.6%) septic patients showed antibacterial resistance at least to one antibiotic. The most common isolates were *Klebsiella pneumonia,* followed by *Escherichia coli and Acinetobacter* species with equal distribution. These isolates were found to be resistant to ampicillin (79.9%), cotrimoxazole (64.1%), and ciprofloxacin (64.1%).

**Conclusions:**

This study revealed a significant problem of antibacterial resistance in the referral hospital. The most commonly prescribed and affordable antibiotics were found to be resistant. So, the hospital and the country as well are expected to minimize the magnitude of the problem through different antimicrobial resistance strategic stewardship initiatives.

## Highlights

• In this study, high prevalence of antibiotic resistance was observed.
• The top resistance bacteria shift from Gram-positive to Gram-negative.
• Commonly prescribed and affordable antibiotics were resistant to the bacteria.
• High number of deaths were observed from patients in the hospital intensive care unit.

## Introduction

Antimicrobial/antibacterial resistance is characterized by a lack of susceptibility to at least one agent in three or more categories of antimicrobials [1]. Antimicrobial resistance (AMR) occurs naturally over time, usually through genetic changes. However, the misuse and overuse of antimicrobials are accelerating this process. In many places, antibiotics are overused and misused in people and animals and are often given without professional oversight [2].

The emergence of AMR is a major public health problem worldwide, particularly in developing countries. The effectiveness of currently available antibiotics is decreasing as a result of increasing resistant strains among clinical isolates. Antibacterial resistance affects hospitalized patients, and the risk is more pronounced in critical patients due to the intensity of the treatment, use of invasive devices, increased risk of transmission, and exposure to antibiotics [3,4].

World Health Organization reported an alarming level of antibacterial resistance with high proportions of resistance to cephalosporins and carbapenems [2]. A similar finding was reported from a study done in a tertiary hospital in India [5]. An increasing trend in antibacterial resistance is also reported from Ethiopia, in which *Escherichia coli, Shigella species, Salmonella species*, and *Staphylococcus aureus* were found to be resistant to commonly prescribed antibiotics, including ampicillin, amoxicillin, penicillin, tetracycline, and trimethoprim/sulfamethoxazole [4].

Various factors have contributed to the rise in AMR, including pressure on health professionals to prescribe antimicrobials [6]. Patient failure to complete their full course of treatment, heavy use of antimicrobials in hospitals, and over-the-counter access to antimicrobials.

The lack of clinical microbiology laboratories to identify the specific etiologic agents and their antimicrobial susceptibility testing has increased empirical therapy, which in turn leads to the emergence of AMR. Moreover, self-antibiotic prescription, lack of access to local antibiogram data, and poor awareness of prescribers about AMR were the leading local factors for AMR development in Ethiopia [7].

Even though there were few studies conducted in Tigray [8,9], up-to-date information is lacking about the prevalence of microbial resistance in hospitalized septic patients. Therefore, the aim of this study is to describe the prevalence of antibacterial resistance among hospitalized patients with sepsis. Taking updated facts is important to identify effective interventions to contain antibiotic resistance. Besides, the current study will also contribute to guiding the rational use of antimicrobials [3]. The findings of this study will be used as input to develop a continuous AMR surveillance system. It will also help to treat physicians to know local patterns of both community and healthcare-associated resistant strains and to choose effective broad-spectrum antibiotics accordingly.

## Methods

### Study area

The study was conducted in Ayder Comprehensive Specialized Hospital (ACSH), a tertiary hospital in Mekelle located 780 km from Addis Ababa, the capital city of Ethiopia. ACSH served more than 9 million people from Tigray and parts of Amhara and afar regions. The hospital has inpatient and outpatient departments, including adult intensive care units and medical wards. There are two adult intensive care units called medical and surgical, with eight beds each. According to the hospital's annual report, the bed occupancy rate was 76.4% [10].

### Study design and period

An institutional-based cross-sectional study design was conducted to describe the prevalence of antibacterial resistance in hospitalized patients.

### Source and study population

Adult patients admitted to inpatient and intensive care with a clinical diagnosis of sepsis.

### Inclusion criteria

Participants above 18 years who are suspected to have sepsis using the quick sequential organ failure assessment (qSOFA).

### Exclusion criteria

Non-bacterial infections (viral, fungal, and protozoal) and participants/caregivers who are not willing to have culture were excluded from the study.

### Sample size determination and sampling technique

The sample size required for the study was estimated using the single population formula.

$$n=\frac{Z\frac{\alpha^{2}}{2}P\left(1-P\right)}{d^{2}}$$

Where:

n = sample size,
Z = is the standard normal deviate, set at 1.96 (for 95% confidence interval),
D = desired degree of precision (taken as 0.05),

P = was taken as 90% of *Klebsiella pneumonia* was resistant to tetracycline [9]. Therefore, based on a single proportion formula including a 10% non-response rate a total of 153 samples were enrolled using consecutive sampling techniques from the hospital. The samples were selected from admitted septic participants to adult intensive care unit (ICU) (medical and surgical) and medical wards.

### Data collection procedure and data quality control

The questionnaire was used to collect data on the socio-demographic characteristics and clinical condition of study participants. A questionnaire that fits to answer the research question of antibacterial magnitude in septic participants was adapted from different literature [2,4,5,7,11]. The socio-demographic and clinical-related data were collected from the participants/caregivers, but the laboratory-related data were collected from laboratory registrations and patient charts. The laboratory samples were collected from both the blood and the foci of infection such as urine, stool, pus, chest, and joint fluids by the hospital health professionals for the description of antibacterial resistance. The training was given to four data collectors and two supervisors with health backgrounds. The investigators also supervised the data collection process throughout the study period.

### Bacterial isolates

Different specimens were collected from both the blood and foci of infection, such as urine, stool, pus, and tracheal aspirate; body fluids (cerebrospinal fluid, pleural, peritoneal, joint, and pericardial fluid) were taken according to the aseptic technique using sterile materials for each sample. A urine sample was collected in the early morning. Collected samples were then transported to the microbiology laboratory of the hospital and cultured as indicated by the treating physician. Identification of bacteria and antimicrobial susceptibility tests were done based on the standard operational procedure of the microbiology laboratory of the hospital. Bacteria were identified based on morphological characters, Gram stain, and biochemical tests. Identification of Gram-positive bacteria was done using gram stain, hemolytic activity on sheep blood agar plates, catalase reaction, and Coagulase test. Gram-negative bacteria were identified based on colony morphology on blood agar and MacConkey agar, followed by biochemical reactions, namely oxidase, triple sugar iron, sulfur indole and motility citrate, lysine decarboxylase, and urease tests.

After bacterial identification, antimicrobial susceptibility tests were done on Mueller-Hinton agar using the Kirby-Bauer disk diffusion method. For *E. coli* and other Enterobacteriaceae: ampicillin (10 μg), ciprofloxacin (5 μg), ceftriaxone (30 ug), and gentamicin (10 μg). For Staphylococcus aureus: penicillin (10 units) and cefoxitin (30 µg). For *Pseudomonas aeruginosa*: ceftazidime (30 µg), ciprofloxacin (5 µg), and gentamicin (10 µg). For Enterococcus species, ampicillin (10 µg) and vancomycin (30 µg) were used. Resistance was interpreted according to Clinical Laboratory Standards Institute (CLSI) guidelines (CLSI, 2017/18) [11].

### Study variables

Dependent variable: antibiotic-resistant level.

Independent variables: socio-demographic variables (age, sex, educational status, marital status, and occupation), clinical characteristics (e.g., co-morbid illness, focus of infection, use of devices, duration of hospital stay), culture-related factors (e.g., type of specimen, timing, and setting of specimen collection).

### Data processing and analysis

Data was cleaned, coded, and entered into Epidata 3.1, then exported to SPSS 22 for analysis. Characteristics of the study participants were analyzed using descriptive statistics such as frequency and percentage for categorical data and median and interquartile range for continuous variables. Bivariate and multivariable logistic regression were done to identify the association between dependent and independent variables, but none of the variables were statistically significant. All the analyzed results were presented with narrative statements complemented with tables and figures.

### Operational definitions

Sepsis: A life-threatening organ dysfunction caused by dysregulated host response to infection [12].

qSOFA- presence of ≥2 parameters listed below;
Low systolic blood pressure (≤100 mm-Hg);
Respiratory rate ≥22 breaths per minute;
Altered mental state (Glasgow coma scale <15) [12,13];

Hospital acquired infection (HAI) - Defined as infections occurring within 48 hours of hospital admission, 3 days of discharge or 30 days of an operation [14].

Community-acquired infection (CAI) - An infection that occurred in the community that doesn't fulfill the criteria for HAI.

Multidrug resistance: Is defined as acquired resistance to at least one antimicrobial agent in three or more antimicrobial categories [1].

## Results

A total of 153 hospitalized participants with a clinical diagnosis of sepsis were included in this study with a 100% response rate. The median age and interquartile range of participants were 30 years (22-49), and the majority of them were male, 112 (73.2%). One hundred forty-seven (96.1%) were Orthodox Christian followers. Regarding their clinical parameters, 86 (56.2%) were admitted to the adult ICU, 114 (74.5%) had documented co-morbid illness, and 128 (83.7%) participants used devices. Furthermore, 54 (35.3%) of the participants died from their illness, and 42 of them were admitted to the ICU (Table 1).

**Table 1:** Socio-economic characteristics and clinical parameter of participants with sepsis admitted in Ayder Comprehensive Specialized Hospital, Tigray, 2020-2021.

Variable	Category	Resistant (%)	Not resistant (%)
Age group in years	<25	24 (35.3)	34 (40)
	25-49	29 (42.6)	29 (34.1)
	≥50	15 (22.1)	22 (25.9)
Gender	Male	54 (79.4)	58 (68.2)
	Female	14 (20.6)	27 (31.8)
Residence (n = 149)	Rural	30 (44.1)	39 (48.2)
	Urban	38 (55.9)	42 (51.8)
Educational status (n = 149)	Not formally attend school	24 (36.9)	31 (37)
	Primary	11 (16.9)	21 (25)
	Secondary	21 (32.3)	16 (19)
	Diploma+	9 (13.8)	16 (19)
Marital status (n = 148)	Single	30 (47.6)	39 (45.9)
	Married	31 (49.2)	42 (49.4)
	Others^a^	2 (3.2)	4 (4.7)
Occupational status (n = 151)	Farmer	08 (12.1)	11 (12.9)
	Government employee	32 (48.5)	32 (37.6)
	Private employee and merchant	09 (13.6)	13 (15.3)
	Daily worker	03 (4.5)	03 (3.5)
	Others	14 (21.2)	26 (30.6)
Admitting ward	Intensive care unit	42 (61.8)	44 (51.8)
	Medical ward	26 (38.2)	41 (48.2)
Associated comorbidity	Yes	54 (79.4)	60 (70.6)
	No	14 (20.6)	25 (29.4)
Availability of device	Yes	58 (85.3)	70 (83.3)
	No	10 (14.7)	14 (16.7)
Outcome of the participants	Improved	39 (60)	51 (64.6)
	Died	26 (40)	28 (35.4)

a Divorced and widowed.

### Participant and healthcare setting factors

The majority of the participants, 110 (71.9%), had previous hospitalizations. Sixty-five (42.8%) of the participants took antibiotics within 90 days of the data collection period. All participants were initiated on empiric antibiotics, and 85 (55.6%) of the participants were made antibiotic revisions as a result of not improving from their infection.

HAI accounted for 87 (58.4%) of the participants. The most commonly identified bacteria in both community and HAIs were *Klebsiella pneumoniae, E. coli, Acinetobacter, Pseudomonas entrobacter, Proteus mirabilis, Proteus vulgaris,* and *Klebsiella oxytoca.*

### Culture and isolated bacteria

**Figure 1:**
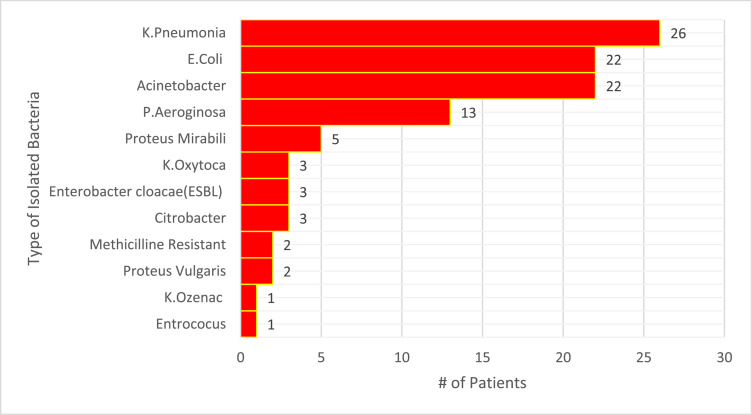
Type of isolated bacteria among participants with sepsis admitted to Ayder Comprehensive Specialized Hospital, Tigray 2020-2021.

In this study, 103 bacteria isolates were identified from urine (39), blood (36), trachea (22), pus (17), stool (04), and sputum (03) samples collected from 70 participants. The results indicated that more than one bacterium was isolated from 18 participants. The most common bacteria that grew from the collected samples was *K. pneumoniae,* which accounted for 26 cases (Figure 1).

**Figure 2:**
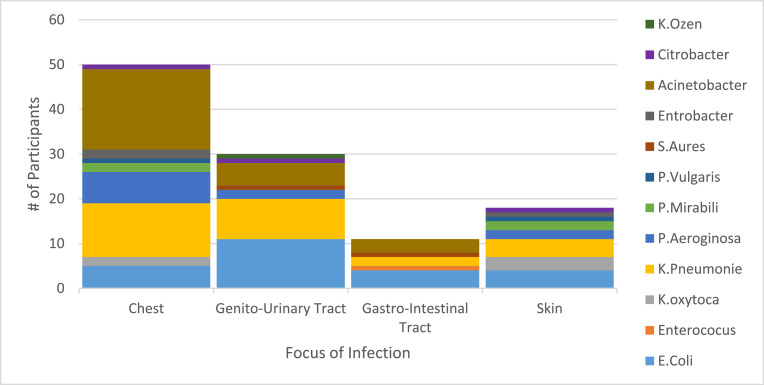
Focus of infection and type of isolated bacteria among septic participants in Ayder Comprehensive Specialized Hospital, Tigray 2020-2021.

More than half of the participants, 83 (54.2%), had a chest focus of infection followed by genitourinary tract infection 38 (24.8%), gastrointestinal 21 (11%), skin 13 (7%), and others 32 (17%). *Acinetobacter, K. pneumoniae*, and *P. aeruginosa* were the top three isolates from the chest focus of infection. Similarly, *E. coli* was the most commonly isolated bacteria from the genitourinary and gastrointestinal tracts focus of infection (Figure 2).

### Magnitude of antibacterial resistance

**Figure 3:**
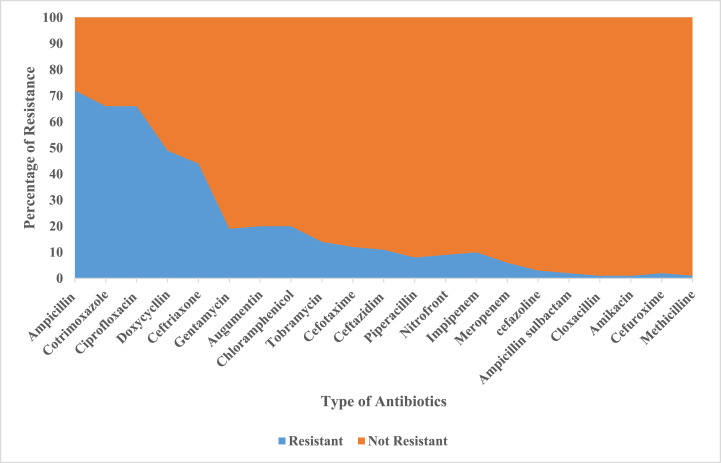
Level of antibiotic resistance among participants with sepsis admitted in Ayder Comprehensive Specialized Hospital, Tigray 2020-2021.

Of the total study participants, 70 (45.8%) had been found infected with antibiotic-resistant bacteria for at least one antibiotic, of which 65 (92.8%) were resistant to two and above antibiotics. The majority of the bacterial isolates showed resistance development against ampicillin (69.9%), cotrimoxazole (64.1%), and ciprofloxacin (64.1%) resistance (Figure 3).

Regarding the resistance of each antibiotic to a specific isolate, *E. coli, K. pneumoniae, Acinetobacter*, and *P. aeruginosa* were found to be the top four bacterial isolates resistant to the top four antibiotics (ampicillin, cotrimoxazole, ciprofloxacin, and ceftriaxone) and susceptible to the top three antibiotics (gentamycin, tobramycin, and imipenem) (Table 2).

**Table 2:** Type of bacteria and resistance level to Antibiotics among participants in Ayder Comprehensive Specialized Hospital, Tigray 2020-2021.

Antibiotics Type	Classification	Escherichia coli	Klebsiella pneumoniae	Pseudomonas aeruginosa	Acinetobacter	Klebsiella oxytoca	Proteus mirabilis	Proteus vulgaris	Enterobacter	Citrobacter	Klebsiella ozenae	Enterococcus	Staphylococcus aureus
		No (%)	No (%)	No (%)	No (%)	No (%)	No (%)	No (%)	No (%)	No (%)	No (%)	No (%)	No (%)
Ampicillin	Susceptible	1 (3.7)	0	0	1 (4.8)	0	0	0	0	0	0	1 (50)	0
	Resistant	26 (96.3)	25 (100)	10 (100)	20 (95.2)	5 (100)	7 (100)	3 (100)	2 (100)	6 (100)	2 (100)	1 (50)	1 (100)
Gentamycin	Susceptible	13 (76.5)	20 (74.1)	5 (41.7)	11 (52.4)	1 (100)	3 (60)	0	2 (66.7)	2 (100)	1 (50)	1 (100)	0
	Resistant	4 (23.5)	7 (25.9)	7 (58.3)	10 (47.6)	0	2 (40)	0	1 (33.3)	0	1 (50)	0	0
Ceftazidime	Resistant	1 (100)	10 (100)	4 (100)	2 (100)	0	0	0	2 (100)	0	0	0	2 (100)
Ciprofloxacin	Susceptible	3 (13)	12 (29.3)	7 (36.8)	6 (18.2)	1 (33.3)	0	0	1 (33.3)	2 (40)	1 (50)	1 (50)	0
	Resistant	20 (87)	29 (70.7)	12 (63.2)	27 (81.8)	2 (66.7)	5 (100)	1 (100)	2 (66.7)	3 (60)	1 (50)	1 (50)	3 (100)
Cefazoline	Susceptible	1 (20)	1 (100)	1 (100)	0	0	0	0	0	0	0	0	1 (100)
	Resistant	4 (80)	0	0	0	0	0	0	0	0	0	0	0
Tobramycin	Susceptible	0	11 (47.8)	4 (33.3)	7 (70)	0	0	0		0	0	0	1 (50)
	Resistant	0	12 (52.2)	8 (66.7)	3 (30)	1 (100)	0	0	2 (100)	0	0	0	1 (50)
Piparacin	Susceptible	2 (100)	5 (100)	1 (100)	1 (16.7)	0	0	0	1 (50)	2 (66.7)	1 (50)	0	
	Resistant	0	0	0	5 (83.3)	1 (100)	1 (100)	0	1 (50)	1 (33.3)	1 (50)	0	1 (100)
Doxycycline	Susceptible	2 (9.5)	2 (7.8)	0	1 (7.1)	0	0	3 (75)	0	0	0	0	1 (50)
	Resistant	19 (90.5)	24 (92.2)	5 (100)	13 (92.9)	2 (100)	4 (100)	1 (25)	2 (100)	4 (100)	0	1 (100)	1 (50)
Chloramphenicol	Susceptible	6 (60)	13 (72.2)	2 (50)	3 (21.4)	1 (50)	0	0		0	0	0	2 (100)
	Resistant	4 (40)	5 (27.8)	2 (50)	11 (78.6)	1 (50)	1 (100)	2 (100)	1 (100)	2 (100)	1 (100)	0	0
Ceftriaxone	Susceptible	2 (10)	1 (6.2)	0	0	0	0	0	0	0	0	0	
	Resistant	18 (90)	15 (93.8)	2 (100)	15 (100)	4 (100)	4 (100)	0	2 (100)	4 (100)	2 (100)	0	1 (100)
Cefotaxime	Resistant	0	10 (100)	4 (100)	5 (100)	0	0	0	1	0	0	0	1
Augmentin	Resistant	2 (100)	11 (100)	3 (100)	5 (100)	0	1 (100)	2 (100)	2 (100)	0	0	0	2 (100)
Imipenem	Susceptible	10 (90.9)	9 (50)	4 (57.1)	5 (45.5)	0	1 (50)	1 (100)	0	0	0	1 (100)	0
	Resistant	1 (9.1)	9 (50)	3 (42.9)	6 (54.5)	0	1 (50)		0	0	0	0	0
Meropenem	Susceptible	0	8 (57.1)	4 (66.7)	1 (25)	0	0	1 (100)	2 (100)	0	0	0	1 (100)
	Resistant	0	6 (42.9)	2 (33.3)	3 (75)	0	0	0	0	0	0	0	
Nitroform	Susceptible	5 (50)	2 (33.3)	0	0	0	0	0	0	0	0	0	1 (50)
	Resistant	5 (50)	4 (66.7)	0	1 (100)	1 (100)	2 (100)	0	0	0	0	0	1 (50)
Cotrimoxazole	Susceptible	4 (17.4)	5 (14.7)	3 (21.4)	2 (7.4)			1 (33.3)	0	0	0	0	1 (33.3)
	Resistant	19 (82.6)	29 (85.3)	11 (78.6)	25 (92.6)	3 (100)	7 (100)	2 (66.7)	2 (100)	4 (100)	1 (100)	1 (100)	2 (66.7)
Methicillin	Resistant	0	1 (100)	0	0	0	0	0				0	
Ampicillin sulbactam	Susceptible	0	0	0	2 (66.7)			0	0	0	0	0	0
	Resistant	0	0	0	1 (33.3)	1 (100)	1 (100)	0	0	0	0	0	0
Cloxacillin	Resistant	0	0	0	0	0	0	0	1 (100)	0	0	0	0
Vancomycin	Susceptible	1 (100)	1 (100)	0	0	0	0	1 (100)		0	0	1 (100)	0
Amikacin	Susceptible	0	4 (100)	1 (100)	2 (100)	0	0	1 (100)	1 (100)	0	0	0	1 (100)

### Associated risk factors for AMR

Variables such as gender, educational status, ward admitted, those who had a skin infection, area of infection acquisition, and having associated comorbidity and availability of devices in the patient were associated with antibiotic resistance in bivariate regression. Even though variables were associated with bivariate regression, no variable was significantly associated in multi-variable analysis (Table 3).

**Table 3:** Associated factors with antibiotics resistance in Ayder Comprehensive Specialized Hospital, Tigray 2020-2021.

Variables	Category	Resistant	Not resistant	Crude odds ratio	*P*-value
Gender	Male	54	58	1.8(0.85-3.78)	0.123
	Female	14	27		
Educational status	Not formally attend school	24	31	1.38(0.52-3.65)	0.521
	Primary	11	21	0.93(0.31-2.78)	0.899
	Secondary	21	16	2.33(0.82-6.63)	0.112
	Diploma+	9	16		
Ward admitted	Intensive care unit	42	44	1.51(0.79-2.88)	0.216
	Medical ward	26	41		
Skin infection	Yes	11	02	8.01(1.71-37.5)	0.008
	No	57	83		
Other infection	Yes	09	23	0.41(0.18-0.96)	0.04
	No	59	62		
Area of acquisition	Community	24	38	0.68(0.35-1.31)	0.247
	Hospital	42	45		
Associated comorbidity	Yes	54	60	1.61(0.76-3.4)	0.215
	No	14	25		
HIV	Yes	02	07	0.31(0.06-1.55)	0.154
	No	51	55		
Cardiac disease	Yes	07	15	0.48(0.18-1.28)	0.141
	No	46	47		
Another comorbidity	Yes	40	37	1.8(0.83-3.93)	0.139
	No	15	25		
Urinary device	Yes	51	47	3.88(1.53-9.79)	0.004
	No	07	25		
Intubation	Yes	38	29	2.82(1.38-5.77)	0.005
	No	20	43		
Tracheostomy	Yes	11	08	1.87(0.7-5.02)	0.212
	No	47	64		

## Discussion

In this study, 187 specimens were collected from 153 (86 from the ICU and 67 from the medical ward) sepsis participants. Seventy (45.8%) participants had positive culture results, all of which indicated antibacterial resistance to at least one antibiotic. The most common bacteria isolated were Gram-negatives (*K. pneumoniae, E. coli, and Acinetobacter),* and the top three antibiotics found to be resistant were ampicillin, cotrimoxazole, and ciprofloxacin. More than 58% of the respondents had hospitalized infections. One hundred three bacteria were isolated from different samples, with urine and blood accounting for 39 and 36, respectively.

In this study, antibacterial resistance was reported in 45.8% of adult participants admitted with suspected sepsis, which is similar to various retrospective studies that have shown an increasing burden [15]. The majority of the isolated bacteria (*E. coli, K. pneumoniae, and Acinetobacter*) showed resistance to ampicillin, cotrimoxazole, and ciprofloxacin and were sensitive to gentamycin, tobramycin, and imipenem. This finding is similar to a study done in India where ciprofloxacin and cotrimoxazole showed decreased susceptibility among the Gram-negatives, but in contrast to our study, they have found a significant number of *K. pneumoniae* resistant to gentamycin [16]. A study done in a tertiary hospital in India among septic patients admitted to ICU reported an alarming level of resistance to cephalosporins from blood and fecal samples isolated (*E. coli*) [17]. Another study from southern Ethiopia and Indonesia also revealed more than 50% resistance observed for ampicillin, cotrimoxazole, penicillin G, chloramphenicol, gentamicin, norfloxacin/ciprofloxacin/levofloxacin, cefotaxime/ceftriaxone/ ceftazidime, erythromycin, and oxacillin [6,18]. In contrast to our findings, the prevalence of at least one resistant Gram-positive and gram-negative organism in a cohort study done among community-acquired septic patients was 13.6 and 13.2%, respectively, in 104 US hospitals [19]. The reason for the discrepancy could be related to a different setup (more developed in this case) and study design, and it was done only in community-acquired sepsis, while this study addresses both community and HAIs.

In this research, the most common bacteria that grew from the culture were *K. pneumoniae, E. coli, Acinetobacter, and P. aeruginosa* for both hospital- and CAIs. A similar finding was reported in India, where *Klebsiella, Pseudomonas*, and *E. coli* were the most common isolates [20,21], and from Indonesia, *Klebsiella, E. coli, and Staphylococcus hominies* were reported with the highest frequency among adult sepsis patients [18,21]. A China study in a burn ward indicated that *Acinetobacter* and *K. pneumoniae* were found in an increasing prevalence [22]. On the contrary, a study done 8 years ago in Ethiopia, Tigray, identified more Gram-positive bacteria [8]. This shift in the dynamics of common isolates might suggest the availability of missed use and overuse of antibiotics in the hospital and the community that requests routine antimicrobial surveillance is mandatory; otherwise, etiologies/isolates can be changed with time and advance of medical practice.

The majority of the study participants had HAIs, with the most common focus of infection being chest, followed by genitourinary. One hundred three bacteria were isolated from urine, blood, tracheal aspirates, and pus samples, and more than 50% of the isolates were from urine and blood samples. This result is in line with the studies done in India, the US, and Ethiopia, where the most common culture-positive samples were urine and blood [8,19,20,23]. Lung infection was the highest focus of infection for septic patients and accounted for deaths in millions associated with resistance [18,21,24].

Despite 71.9% having a history of hospitalization and 42.8% receiving antibiotics within 90 days of the data collection period, all participants started empiric treatment during the current admission without taking culture. As a result, antibiotic revisions were made in 55.6% of them because they did not show any improvements with the empiric course. This finding is similar to a study done in Ethiopia and Turkey [[25], [26], [27]]. This may be associated with the level of health professionals’ knowledge, attitude, and practice.

The results of this study have important implications. In this review, 45.8% of septic patients were found to have antibacterial resistance. The common bacterial isolates found were gram-negative regardless of the area of acquisition and/or the focus of infection. This is a major epidemiological shift where the commonest etiologies some 8 years back in this region were mainly Gram-positive isolates [8]. This is a clear indication that there is a transition of etiologies, and health professionals should be updated about the local resistance patterns otherwise we may fail in controlling the transmission of resistant bacteria. It is also crucial that continuous surveillance and antimicrobial stewardship programs be established and integrated into the health system.

### Limitations of the study

Due to the COVID-19 pandemic followed by the war and siege in Tigray, there were supply interruptions in the microbiology laboratory; all the antibiotics were not checked, and the data collection was interrupted in between for a few months. Similarly, the calculated sample size was small and difficult to generalize to the similar setup settings.

## Conclusion

The current study showed a significant magnitude of antibacterial resistance in hospitalized patients with sepsis. The commonest bacteria isolated were gram-negative in both hospital and CAIs. In addition, it also revealed that the most commonly prescribed and affordable antibiotics were found to be resistant. Hence, this study will enrich the hospital to establish and/or activate a strong and functioning antimicrobial stewardship program that can run a continuous antimicrobial surveillance system and have its local antibiogram. This also enables healthcare professionals to have clear and up-to-date information about the common isolates that help them choose the right empiric antibiotics for their patients.

## Declarations of competing interest

The authors have no competing interests to declare.
